# Repetition Hampers Flexible Object Manipulation Under Visual Uncertainty

**DOI:** 10.1111/ejn.70155

**Published:** 2025-06-17

**Authors:** Catherine Anne Sager, Ian Greenhouse, Michelle Marneweck

**Affiliations:** ^1^ Department of Human Physiology University of Oregon Eugene Oregon USA; ^2^ Institute of Neuroscience, University of Oregon Eugene Oregon USA; ^3^ Phil and Penny Knight Campus for Accelerating Scientific Impact Eugene Oregon USA

**Keywords:** anterograde interference, motor control, repetition, visual uncertainty

## Abstract

Seemingly simple actions, like reaching for and lifting an object, involve the coordination of distinct neural pathways within the dorsal and ventral streams. These components can be differentially affected by repetition‐induced anterograde interference, where extensive practice on one task impairs performance on subsequent tasks. Repetition leads to rigid movement patterns, making it harder to adapt flexibly to new situations, especially in tasks with sensory uncertainty that require the brain to rely more on past experiences (i.e., sensorimotor memories). To explore this, we tested whether object‐use tasks, which depend on the ventral stream, are more affected by this interference than a simpler reach‐to‐button task with helpful visual cues. Participants completed two tasks: a reach‐to‐button task involving pressing buttons on either side of a symmetrical object and an object‐use task where the same object had a hidden, asymmetric center of mass (CoM). To measure interference, we manipulated how many times participants lifted the object with the weight on one side before switching it to the other side. Our results showed that interference was strongest in the object‐use task, where uncertain visual information forced participants to rely on sensorimotor memories. In contrast, the reach‐to‐button task, supported by helpful visual cues, showed no significant interference. This suggests that tasks relying on the ventral stream are more vulnerable to interference, particularly when sensory feedback is unclear. Our findings highlight how repetition affects different movement types and emphasize the need for a balance between repetition and flexibility in motor learning.

## Graphical abstract

Repetition‐induced interference was magnified in object‐use tasks under visual uncertainty but absent in visually guided reaching. Together with prior work, these findings suggest that repetition hampers flexibility in both dorsomedial‐ and dorsolateral‐guided actions requiring ventral stream input, highlighting how repetition challenges flexible control of prehensile actions under conditions of heightened visual uncertainty.

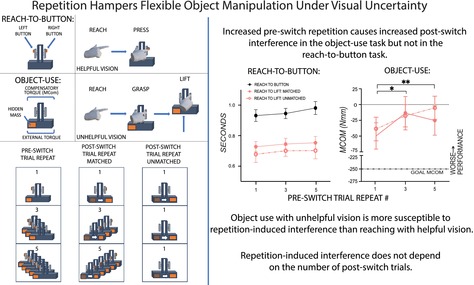

**Abbreviations**
AIP: anterior intraparietal area
CoM: center of mass
COPdiff: center of pressure difference
GFdiff: grip force difference
LFdiff: lift force difference
MIP: medial intraparietal area
N: newton
N mm: newton millimeters
PMd: dorsal premotor cortex
PMv: ventral premotor cortex

## 1 Introduction

Prehensile actions, like reaching, grasping, and manipulating objects, rely on separate yet coordinated processes (Jeannerod 1984). These actions are vulnerable to repetition‐induced anterograde interference, where extensive practice on one task makes it harder to perform a new one (Sing and Smith 2010; Lerner et al. 2020). This effect has been observed in both reaching (Miall et al. 2004; Sing and Smith 2010; Verstynen and Sabes 2011; Leow et al. 2014; De La Fontaine et al. 2023) and reach‐to‐lift tasks (Fu and Santello 2012; Fu and Santello 2015; Sager et al. 2024). Since repetition‐induced anterograde interference has been observed in reach and object manipulation tasks, it suggests that these different components of prehensile action, as well as the neural circuitry that guide them, are similarly affected by this phenomenon.

The different components of prehensile actions are planned by partly separate neural pathways in the dorsal stream, which guides actions, and the ventral stream, which processes object perception. Reaching requires transforming visual information about an object's location into a motor command and is guided by the dorsomedial stream, involving the visual area V6A, the medial intraparietal area (MIP), and the dorsal premotor cortex (PMd) (Vesia et al. 2017; Gallivan and Goodale 2018). Grasping and complex manipulations necessary for lifting or interacting with objects require integrating visual information about the object's properties to guide hand configuration and forces and are facilitated by a dorsolateral stream connecting the anterior intraparietal area (AIP) and the ventral premotor cortex (PMv) (Fattori et al. 2010; Gallivan and Goodale 2018). Additionally, it is becoming increasingly clear that the ventral stream is critical for complex manipulative actions, especially when overt visual cues (e.g., object shape) are unreliable and conflict with covert cues (e.g., object's weight distribution) learned from previous experiences (Gallivan et al. 2014; Marneweck et al. 2018; Klein et al. 2019; Marneweck and Grafton 2020a, 2020b, 2020c).

While previous studies suggest that both reaching and object manipulation tasks are affected by repetition‐induced anterograde interference, another hypothesis is that any part of prehensile action can be impacted by interference when faced with visual uncertainty that necessitates increased reliance on ventral stream input. Consistent with this, anterograde interference has been predominantly observed in complex motor learning tasks that involve force‐field motor adaptation (Sing and Smith 2010) and visuomotor rotation adaptation (Lerner et al. 2020; Hamel et al. 2021) and in object manipulation tasks that require precision in lifting visually symmetrical objects with asymmetrical mass distributions (Salimi et al. 2000; Sager et al. 2024). These tasks share two common factors: discordant visuomotor mapping and limited helpful visual information, necessitating greater reliance on prior knowledge and ventral stream input, which have been shown to play a crucial role in the memory of task and object properties (Gallivan et al. 2014). Thus, motor tasks that depend heavily on ventral stream processing, particularly under conditions of visual uncertainty, are susceptible to anterograde interference.

**FIGURE 1:**
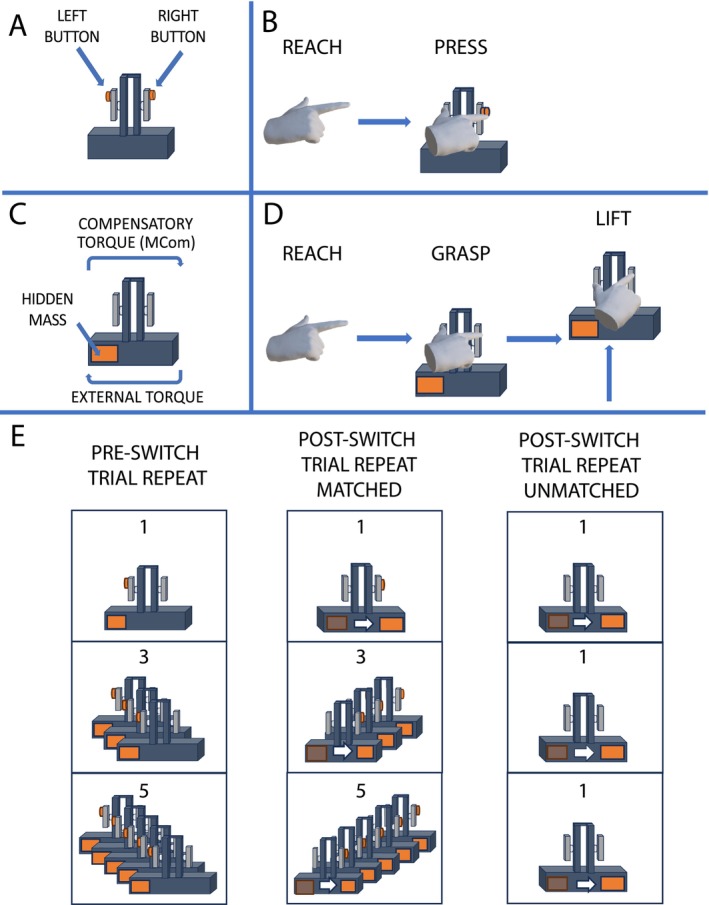
Object and task design. Participants performed two tasks: (A,B) reaching to press a left‐ or right‐sided button or (C,D) reaching, grasping, and lifting an object with a left‐ or right‐sided center of mass (CoM) to minimize tilt. To examine repetition‐induced anterograde interference, we varied the number of pre‐switch trials (1, 3, or 5) before switching the button side or CoM side (E, left and middle panels). We compared this design to that of Sager et al. (2024), which had only one post‐switch trial, to assess whether the number of post‐switch trials influenced interference effects (E, right panel).

Here, we test the hypothesis that visual uncertainty modulates the susceptibility of the different components of prehensile actions to repetition‐induced anterograde interference. To test this hypothesis, participants completed two tasks (Figure 1). For the reach‐to‐button task, participants reached and pressed a button on either side of a symmetrically shaped object. For the object‐use task, participants reached, grasped, and lifted the same symmetrically shaped object that had a hidden asymmetric mass distribution to minimize its tilt (Salimi et al. 2000; Santello and Soechting 2000; Marneweck et al. 2018; Bland et al. 2023). We quantified the effects of repetition‐induced anterograde interference by selectively varying the number of button presses or object lifts with the button or weight on a given side before switching it to the other side. Visual information was unhelpful for the object‐use tasks, requiring predominant reliance on prior experience. In contrast, visual information was helpful to the reach component in both reach‐to‐button and object‐use tasks. Our results are in line with the hypothesis that repetition‐induced anterograde interference, present in the object‐use component and not in the reach components, arises under conditions of heightened visual uncertainty, depending on prior knowledge and ventral stream processing during early sensorimotor learning.

## 2 Materials and Methods

Participants completed a reach‐to‐button task (Figure 1A,B) and an object‐use task (Figure 1C,D). In the reach‐to‐button task, participants used their right index finger or thumb to press a button on the right or left, respectively, of a symmetrically shaped object. In the object‐use task, participants were instructed to reach, grasp, lift, and minimize tilting an inverted T–shaped object with a concealed off‐centered mass. Success in the reach‐to‐button task involves pressing the correct button, whereas success in the object‐use task requires planning and generating a compensatory torque at lift onset (i.e., anticipatory force control) through a combination of digit position, grip, and lift forces. In both tasks, we manipulated the number of trials in which participants were to reach to a given side or to lift an object with a given CoM before we switched the reach or CoM side. By selectively varying the pre‐switch trial number and measuring performance outcomes on the trial immediately following the switch, we evaluated the switch cost (i.e., presence of repetition‐induced anterograde interference) of repeatedly planning context‐specific reach and object‐use actions, respectively (Figure 1E). Secondarily, we compared whether repetition‐induced anterograde interference is affected by the familiarity with a switched plan, by comparing results from this dataset (with matched pre‐ and post‐switch trials) with a previously published study showing repetition‐induced anterograde interference with unmatched pre‐ and post‐switch trials (Sager et al. 2024).

### 2.1 Participants

Twenty healthy right‐handed young adults (median age: 25, range: 18–35, standard deviation = 5; 13 females) participated in this study. The study used a within‐subject design for the reach‐to‐press and reach‐to‐lift tasks. Additionally, we performed a comparative analysis with the reach‐to‐lift cohort and another reach‐to‐lift cohort from a previous study (*N* = 30, M = 23, SD = 5; 18 females) (Sager et al. 2024). The studies and all their procedures were approved by the University of Oregon Institutional Review Board, and all participants gave written informed consent.

### 2.2 Apparatus

For the reach‐to‐button task, we connected two button switches to the object with a custom response board (Makey Makey v.1.2, JoyLabz) for accurate button press and timing. For the object‐use task, a three‐camera motion‐tracking system (Precision Point Tracking System, WorldViz) with a frame rate of 150 fps (camera resolution: 1280 × 1024 VGA) was used to track the vertical height of the object. The system's spatial accuracy within a 3 × 3 × 3 m volume was ≤ 1 mm. Two near‐infrared LED markers were affixed securely to the covers on the horizontal base of the object to monitor the vertical position of the object. Grip forces and torque that were applied to the grip surfaces of the object were recorded at a frequency of 500 Hz through force/torque transducers (Mini27 Titanium, ATI Industrial Automation, NC). These force transducers were attached between each grip surface and the vertical column of the T‐shaped object. Grip force, load forces, and torque, with resolutions of 0.03 N, 0.015 N, and 0.375 N mm were measured by the transducers. Data were filtered using a fourth‐order low‐pass Butterworth filter, applying a cutoff frequency of 5 Hz. The T‐object was 3D‐printed with chopped carbon fiber containing nylon (Onyx, Markforged). The object's vertical column (height: 9.0 cm; width: 5.0 cm; depth: 3.2 cm) had smooth elongated grasp surfaces (height: 7.4 cm; width: 4.5 cm; depth: 0.8 cm; between‐grasp distance: 8.2 cm) with buttons (height: 2.0 cm, width: 2.0 cm, depth: 0.6 cm) attached on either side on flat elongated surfaces (height: 7.4 cm; width: 4.5 cm; depth: 0.8 cm; between‐grasp distance: 8.8 cm) or ridged elongated grasp surfaces attached on either side (height: 7.4 cm; width: 4.5 cm; depth: 0.8 cm; between‐grasp distance: 8.2 cm). The depth dimensions of the grasp surfaces were marginally greater than the diameter of the transducer surfaces (limiting the opportunity to cause torque in a yaw direction). A lead cylinder (height: 4.5 cm; diameter: 3.8 cm; mass: 490 g) was concealed in the horizontal base (height: 5.6 cm; width: 4.9 cm; depth: 18.3 cm). The total mass of the object was 936 g with an external torque of 250 N mm.

### 2.3 Experimental Procedures

Participants were seated in a chair at a comfortable height such that their right forearm and upper arm made a right angle while resting on the table. They were instructed to keep their left arm from resting on the table. At the start of each trial, participants pressed a keyboard button (home button) with their right index finger, 20 cm from the T‐shaped object that they would interact with in both the reach‐to‐button task and the object‐use task. A “left” or “right” audio cue informed them which button to press in the reach‐to‐button task and the heavier side in the object‐use task. A beep occurred 1.25 s after the left/right side audio cue instructed participants to reach and press the left/right side button (Figure 1B) or to lift the object without tilting it at all times (Figure 1D). In the reach‐to‐button task, participants were required to press the indicated button with one of their digits, while the digit on the other side rested below its respective button; i.e., when participants were hitting the right button with their right index finger, the thumb would rest below the left button and vice versa. In the object‐use task, participants were informed that they could grasp the object anywhere along the elongated grip surfaces. The object was lifted to a height marker (11 cm). A second beep (2.50 s after the first beep) instructed participants to release the object and return their right index finger to its start position on the keyboard button. Participants were told to complete both tasks at a natural pace and to attempt to lift the object without tilting it to the weighted side at all times. Both tasks were completed in a single session in counterbalanced order between subjects. Which of the two buttons to press first and whether CoM started on the left or right side were also counterbalanced between subjects. Nevertheless, there were no significant differences in reach kinematics on the reach‐to‐button task or torque performance on the object‐use task between groups who completed the reach‐to‐button task first versus those who completed the object‐use task first (all *p*s > 0.05).

To examine the effects of repetition on anterograde interference, we manipulated the number of trial repeats in both the reach‐to‐button and object‐use tasks before implementing a switch in the reach direction or object CoM (Figure 1E). For the object‐use task, we varied the number of lifts with the object CoM on a given side before switching it to the other side. For the reach‐to‐button task, we manipulated the number of reach trajectories to a given side before switching it to the other side. In both tasks, participants were exposed to five sets of one, three, and five pre‐ and post‐switch trials, giving a total of 91 trials (45 pre‐switch presses/lifts and 46 post‐switch presses/lifts). One‐minute breaks were provided after every 20 trials.

As a secondary goal in the object‐use task, we determined if repetition‐induced anterograde interference was modulated by familiarity with the pre‐ and post‐switch action plans. Specifically, we matched pre‐ and post‐switch trial numbers to test whether the observed interference previously shown (Sager et al. 2024) could be attributed to unequal experience with the pre‐ and post‐switch action plans. Here, participants were similarly familiar with pre‐ and post‐switch trials, experiencing the same number of repetitions on either side of the switch. In contrast, pre‐ and post‐switch trials were not matched in a previous study (Sager et al. 2024) in which we observed repetition‐induced anterograde interference in the object‐use task, with one post‐switch trial, regardless of pre‐switch trials (45 pre‐switch lifts and 15 post‐switch lifts for a total of 60 trials). We compared the effects of repetition‐induced anterograde interference between these datasets with a matched number of pre‐ and post‐switch trials (Figure 1E, middle panel) and an unmatched number of pre‐ and post‐switch trials (Figure 1E, right panel). By equally balancing the number of pre‐ and post‐switch trials in the matched condition, we controlled for potential differences in familiarity with the post‐switch plan.

### 2.4 Data Processing

The reach components on the reach‐to‐button and object‐use tasks are as follows:

1. Reach phase is defined as the time from releasing the keyboard button to when the subject presses the button in the reach‐to‐button task or grasps the object with more than 0.2 N of grip force in the object‐use task.
2. Reaction time is defined as the time from the movement cue to releasing the keyboard button in both tasks.

The grasp‐to‐lift components on the object‐use task are as follows:

1. The load phase is defined as the interval from when the net lift force first exceeds 0.2 N and continues to increase consistently for the next 20 consecutive samples.
2. To quantify grip–lift force coupling, we measured the coordination between the change rate of the grip force (GF_rate_) and the change rate of the lift force (LF_rate_). We used a cross‐correlation approach, shifting the grip force rate in 2.5‐ms time steps relative to the lift force rate over a 200‐ms window (i.e., from 100 ms before to 100 ms after the lift force rate), computing the correlation at each shift. The peak correlation value across these shifts was used as our measure of grip force–lift force coupling (GF‐LF coupling) (Hermsdörfer et al. 2003; McDonnell et al. 2006).
$$r\left(\tau \right)=\text{corr}\left({\mathrm{GF}}_{\text{rate}}\left(t+\tau \right),{\mathrm{LF}}_{\text{rate}}\left(t\right)\right)$$
  where *r*(*τ*) is the correlation coefficient at time lag *τ*, and the maximal *r*(*τ*) across all lags was used as the trial‐level measure of GF–LF coupling.

The lift components on the object‐use task are as follows:

1 Grip force mean (GF_mean_) at lift onset is the instantaneous average in grip force of each digit in newtons. This was calculated using a numerical averaging method:
$${\mathrm{GF}}_{\text{mean}}=\left({\mathrm{GF}}_{\text{thumb}}+{\mathrm{GF}}_{\text{index}}\right)/2$$

2 Lift force difference (LF_diff_) at lift onset is the variation in the tangential component of the force produced by each digit (N).
$$\text{Lift force difference}\;\left({\mathrm{LF}}_{\text{diff}}\right)={\mathrm{LF}}_{\text{thumb}}-{\mathrm{LF}}_{\text{index}}$$
  A higher thumb than index finger lift force shows positive values, and a higher index finger than thumb lift force shows negative values. Larger differences indicate a more asymmetric lift force–sharing pattern, whereas a zero value indicates a symmetric lift force–sharing pattern.
3 Center of pressure (COP) at lift onset is the measure of digit position defined as the point of contact of each digit on the grip surface relative to the center of the transducer (in millimeters). This was computed using the following formula:
$${\mathrm{COP}}_{\text{digit}}=\left({\mathrm{Tx}}_{\text{digit}}-\left({\mathrm{LF}}_{\text{digit}}\times \text{grip surface thickness}\right)\right)/{\mathrm{GF}}_{\text{digit}}$$
  where *Tx*_digit_ is the digit torque in the frontal plane (N mm). The difference between thumb and index finger placement was used to identify grip configuration:
$$\text{Center of pressure difference}\;\left({\mathrm{COP}}_{\text{diff}}\right)={\mathrm{COP}}_{\text{thumb}}-{\mathrm{COP}}_{\text{index}}$$
  Positive values indicate higher thumb than index finger COP, and negative values indicate higher pointer finger than thumb COP. Larger differences indicate a more asymmetric, noncollinear grip configuration, whereas a zero value indicates a symmetric, collinear grip configuration.
4 Compensatory moment or torque (M_Com_) at lift onset is the anticipatory torque generated by the digits measured in newton millimeters to counter the external torque of the object. This was computed using the following formula:
$$M_{\mathrm{Com}}=\left({\mathrm{LF}}_{\text{diff}}\times \left(d/2\right)\right)+\left({\mathrm{GF}}_{\text{mean}}\times {\mathrm{COP}}_{\text{diff}}\right)$$
  where *d* is the width between both grip surfaces (8.20 cm). A positive *M*_Com_ indicated a clockwise moment, and a negative *M*_Com_ indicated a counterclockwise moment.

For participants who began the task with the CoM on the right, *M*_Com_, LF_diff_, and COP_diff_ values were multiplied by −1 to avoid the statistical complication caused by different signs of *M*_Com_ when manipulating an object with a left vs. right CoM (Sager et al. 2024).

### 2.5 Data Analyses

Bayesian two‐way ANOVA analyses were performed to assess the effects of CoM (left vs. right) and trial repetition number (one vs. three vs. five) on lift performance. The primary goal was to determine whether the CoM position (left vs. right) affected lift performance, specifically to justify that a post‐switch lift could be considered equivalent regardless of CoM side. The analysis showed that differences in CoM position and trial repetition did not significantly influence lift performance, supporting our assumption of equivalency across CoM positions for post‐switch lifts. By multiplying by −1, a positive *M*_Com_ that nears or matches the object's external torque on pre‐switch trials exhibits successful torque generation, resulting in minimal roll.

Our analyses focused on identifying the effect of repetition‐induced anterograde interference on reach‐to‐button and object‐use tasks. Additionally, we evaluated whether this interference was modulated by familiarity (i.e., lifting experience) with the adjusted, post‐switch action plan. For the reach component, we ran two 2 × 3 ANOVAs to examine the effects of pre‐switch trial repetition (one vs. three trials and one vs. five trials) and condition (reach to button, object use matched, and object use unmatched). For the object‐use component, we conducted a 2 × 2 ANOVA with pre‐switch trial repetition (one vs. three and one vs. five) and familiarity (matched vs. unmatched pre‐ and post‐switch trials) as factors, followed by Bonferroni‐corrected pairwise comparisons to adjust for multiple comparisons and identify specific significant differences between trial repetition conditions. Outcome measures on the reach component were reaction time and reach phase in both reach‐to‐button and object‐use tasks. Outcome measures on the grasp component of the object‐use task were load phase and GF–LF coupling. Outcome measures on the lift component of the object‐use tasks were *M*_Com_ and its goal‐oriented components (LF_diff_ × (grip width/2); GF_mean_ × COP_diff_).

## 3 Results

### 3.1 No Repetition‐Induced Anterograde Interference in the Reach Component of the Reach‐to‐Button and Object‐Use Tasks

**FIGURE 2:**
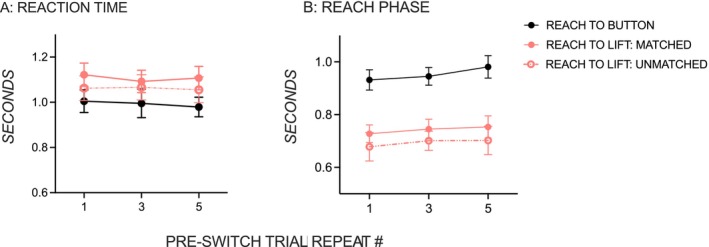
(A) Reaction time and (B) reach phase. Mean reaction time and reach phase on post‐switch trials on the reach‐to‐button task (solid black line, closed circles) and object‐lift tasks with matched pre‐ and post‐switch trials (solid pink line, solid circles) and with unmatched pre‐ and post‐switch trials (dashed pink line, open circles). Error bars are ± 1 standard error.

Figure 2 shows that there was no evidence of repetition‐induced anterograde interference on the reach component of prehensile action with or without the subsequent object‐use component. Reaction times were similar on post‐switch trials, whether they followed one, three, or five pre‐switch trials (Figure 2A), in both the reach‐to‐button and the object‐use tasks (*p'*s > 0.05). Furthermore, the effects of repetition‐induced anterograde interference were similar between object‐use tasks with matched and unmatched pre‐ and post‐switch trials, giving no effect of familiarity or interaction (*p'*s > 0.05). Similar to the reaction time results, reach phases on post‐switch trials (Figure 2B) were unaffected by whether they followed one, three, or five pre‐switch trial repeats (*p'*s > 0.05) in all tasks, again showing no familiarity effect or interaction (*p'*s > 0.05).

### 3.2 No Repetition‐Induced Anterograde Interference in the Grasp‐to‐Lift Component of the Object‐Use Task

#### 3.2.1 Load Phase

**FIGURE 3:**
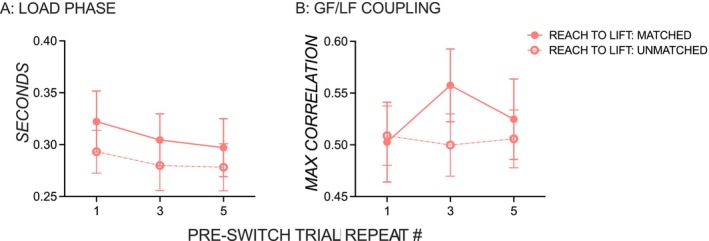
(A) Load phase and (B) the difference between grip and lift force initiation. Mean load phase and grip force–lift force coupling on post‐switch trials on the object‐use tasks with matched (solid pink line, solid circles) and unmatched (dashed pink line, open circles) pre‐ and post‐switch trials. Error bars are ± 1 standard error.

Figure 3A shows no repetition‐induced anterograde interference on the load phase in the object‐use task irrespective of whether the number of post‐switch trials matched or mismatched with the number of pre‐switch trials. The number of pre‐switch lifts did not affect the load phase on post‐switch trials in the reach‐to‐button or the reach‐to‐lift task (*p'*s > 0.05), and there was no effect of familiarity and no interaction (*p'*s > 0.05).

#### 3.2.2 Difference Between Grip and Lift Force Initiation Time

Figure 3B shows that increasing pre‐switch trial repeats did not show repetition‐induced anterograde interference on the GF–LF coupling of post‐switch trials (*p'*s > 0.05). There was no effect of familiarity and no interaction (*p'*s > 0.05).

### 3.3 Presence of Repetition‐Induced Anterograde Interference in the Lift Component of the Object‐Use Tasks

**FIGURE 4:**
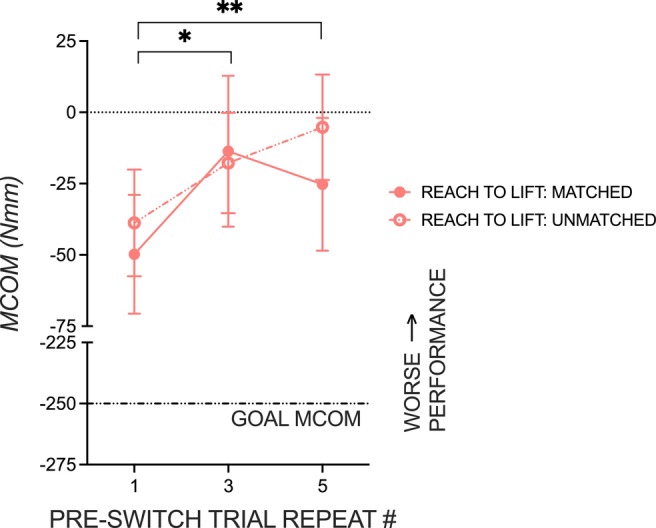
Effect of pre‐switch trial repeat on post‐switch performance. Repetition‐induced anterograde interference on performance, quantified as the group mean compensatory torque (*M*_Com_), in object‐use tasks with matched pre‐ and post‐switch trials (solid pink line, solid circles) and with unmatched pre‐ and post‐switch trials (dashed pink line, open circles). Statistical significance is shown with an asterisk (*p < 0.05, **p < 0.001). Error bars are ± 1 standard error.

Figure 4 shows that anterograde interference is magnified in post‐switch trials when pre‐switch trial repeats are increased. Moreover, these results extend previous work by identifying that anterograde interference is present whether the number of post‐switch trials matched or mismatched with the number of pre‐switch trials, further showing the impact of repetition on performance in object‐use tasks. Figure 4 shows decreased behavioral performance on the trial following a CoM switch as a function of increasing the number of pre‐switch trial repeats. The effect of pre‐CoM switch trial repetition on *M*_Com_ following the CoM switch was significant, *F*(1, 48) = 9.90, *p* = 0.0028, *η*_p_^2^ = 0.17, in object‐use tasks with matched and unmatched pre‐ and post‐switch trials, giving no effect of familiarity or interaction (all *p'*s > 0.05). Bonferroni‐corrected pairwise comparisons revealed significant differences in performance between one and three trial repeats before the switch (*p* = 0.017) and one and five trial repeats before the switch (*p* = 0.007), whether the number of post‐switch trials was matched or mismatched.

Since we identified repetition‐induced anterograde interference in *M*_Com_, which suggests that repeated trials influenced participants' motor strategies when interacting with the object, we decided to examine the effect of pre‐switch repetition and familiarity (and their interaction) on goal‐oriented torque components of *M*_Com_. This was done to better understand which specific aspects of *M*_Com_ are driving the interference effect.

#### 3.3.1 Lift force torque component

**FIGURE 5:**
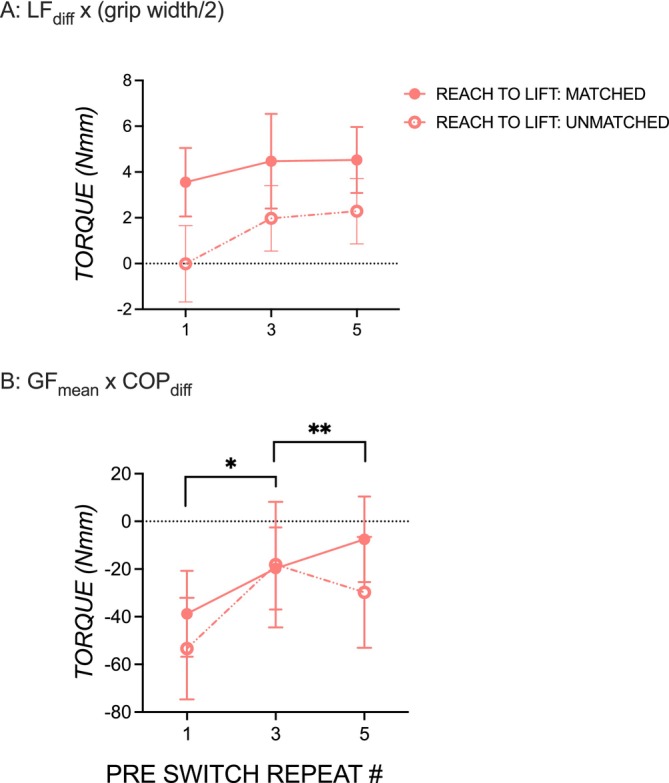
Effect of pre‐switch trial repeat on contributors of compensatory torque on post‐switch trials. Post‐switch digit lift force partitioning by object grip width (A, LF_diff_ × grip width/2) and grip force by digit positioning (B, GF_mean_ × COP_diff_) in object‐use tasks with matched pre‐ and post‐switch trials (solid pink line, solid circles) and with unmatched pre‐ and post‐switch trials (dashed pink line, open circles). Statistical significance is shown with an asterisk (*p < 0.05, **p < 0.001). Error bars are ± 1 standard error.

Figure 5A shows that the torque component generated by the product of LF_diff_ and half of the grip width remained relatively stable across post‐switch trials, regardless of whether participants experienced one, three, or five pre‐switch trials. Positive values indicated that participants tended to replicate their pre‐switch lift force strategy, and while this tendency increased slightly with more pre‐switch trials, the effect was not statistically significant (*p'*s > 0.05). There were no significant effects of familiarity or interaction (*p'*s > 0.5).

#### 3.3.2 Grip force x Digit position torque component

Figure 5B shows that the torque component generated by the product of GF_mean_ and COP_diff_ significantly increased as a function of pre‐switch trial repeats in both object‐use tasks with matched and unmatched pre‐switch trials. There was a significant effect of pre‐switch trial repeat on GF_mean_ × COP_diff_, *F*(1, 48) = 9.30, *p* = 0.0037, *η*_p_^2^ = 0.16, with no effect of familiarity or interaction (all *p*s > 0.05). Bonferroni‐corrected pairwise comparisons revealed significant differences in GF_mean_ × COP_diff_ between one and three trial repeats before the switch (*p* = 0.013) and one and five trial repeats before the switch (*p* = 0.0037), whether the number of post‐switch trials was matched or mismatched.

The repetition‐induced anterograde interference observed in *M*_Com_ appears to be driven by a combination of *M*_Com_ components rather than a single component. The worsening trend in LF_diff_ scaled by grip width, following increased pre‐switch repetitions, suggests that the thumb and index finger lift forces are not adapting effectively to the weight shift, leading to inefficient lift force generation. COP_diff_ scaled by GF_mean_ was more strongly affected; as the number of pre‐switch repetitions increased, participants showed greater difficulty adjusting their digit positions to the new torque demands. The cumulative effect of these factors likely contributes to the observed repetition‐induced anterograde interference in *M*_Com_.

## 4 Discussion

In this study, we investigated how repetition‐induced anterograde interference manifests in different components of prehensile actions. Consistent with our hypothesis, repetition‐induced anterograde interference predominantly affected the lift component of an object‐use task, which required manipulating a symmetrically shaped object with a hidden asymmetrical mass. In contrast, no significant interference was observed in the reaching component where visual information about the targets was available to guide the action. These results indicate that repetition‐induced anterograde interference is more likely to emerge in object‐use tasks, particularly when visual information is insufficient or incongruent with a key object property. Importantly, this effect could not be explained by differences in familiarity with the post‐switch condition. Performance on the first post‐switch trial did not differ between matched and unmatched conditions, indicating that the interference effect could not be explained by reduced experience or practice with the post‐switch action plan.

The main results described here align with previous findings that prehensile reach tasks with discordant visuomotor mapping, such as those in visuomotor rotation and force‐field adaptation tasks, are susceptible to repetition‐induced anterograde interference (Sing and Smith 2010; Loh et al. 2010; Lerner et al. 2020; Hamel et al. 2021; De La Fontaine et al. 2023). However, visuomotor and force‐field adaptations, as well as their aftereffects, typically occur over longer timescales than the repetition‐induced hampering effects that were observed in our object‐use task. The apparent alignment between these phenomena may reflect parallel mechanisms that operate over different timescales. While interference can persist over long durations in adaptation tasks, repetition‐induced anterograde interference in our study likely reflects a short‐term disruption rather than a savings‐like effect (James et al. 2022; Zhou et al. 2022). Our results extend prior work by showing, first, that object lifting, like reaching, is similarly sensitive to repetition‐induced anterograde interference under conditions of heightened visual uncertainty. This effect does not depend on the number of post‐switch lifts that matched or varied with the number of pre‐switch trials. Second, visually guided reaching is not affected by repetition‐induced anterograde interference under conditions of visuomotor congruence. Together, these results suggest that repetition‐induced anterograde interference effects on dorsal and ventral streams for visually guided actions depend on the presence or absence of useful visual information.

Our findings highlight that repetition, while potentially beneficial for reinforcing motor behaviors in certain contexts, is maladaptive in tasks that require flexibility, particularly when visuomotor incongruency is present, i.e., driving in foggy conditions, using mirrors (i.e., under conditions where visual information is reversed), walking on a treadmill with a screen (i.e., the environment could move at a different speed than one's actual movements). When visual information is unhelpful, achieving task success, for example, requires relying predominantly on sensorimotor memories associated with previous experiences (Salimi et al. 2000; Zhang et al. 2010; Baugh et al. 2012; Schneider and Hermsdörfer 2022). Our results and those of others suggest that in the presence of unreliable visual feedback, repetition induces sensorimotor memories that are resistant to change and overgeneralized, translating to stereotyped motor behaviors that challenge the ability to switch between motor plans in variable environments (Salimi et al. 2000; Zhang et al. 2010; Fu and Santello 2012). These findings are especially relevant to older populations, where stereotyped behaviors are more common at multiple levels of the motor system (Holl et al. 2015; Schneider et al. 2019; Cassady et al. 2020; Wittenberg et al. 2022) and may impede the ability to adapt to new or conflicting sensory information (Wolpe et al. 2020; Sager et al. 2024). In these cases, training stereotypy may be counterproductive, limiting the ability to adjust motor plans to suit changing environmental demands. The lack of repetition‐induced anterograde interference in the reach‐to‐button task with helpful vision information suggests that it is the incongruence between vision and motor output that exacerbates interference, which can affect any component of prehensile action requiring motor adjustments.

Our results and that of others suggest that both dorsal and ventral streams, responsible for reach and object manipulation, are susceptible to repetition‐induced anterograde interference in the presence of sensory uncertainty. That said, repetition‐induced anterograde interference can be induced under conditions other than sensory uncertainty, suggesting additional factors that modulate its susceptibility. Fu and Santello (2012) demonstrated that manipulating L‐shaped objects with helpful visual cues induced anterograde interference. Similarly, interference in torque compensation tasks persists even when helpful geometric cues are present (Schneider et al. 2020), although its magnitude is reduced compared to when such cues are absent (Schneider et al. 2019). These findings suggest that while sensory uncertainty can exacerbate interference, object manipulation and its neural control are susceptible to interference irrespective of visual incongruence.

In addition to the role of visual congruency and, potentially, dorsolateral and ventral involvement during object manipulation, Verstynen and Sabes (2011) provided evidence that there are additional factors that may contribute to susceptibility to anterograde interference. Their reach‐to‐target task with accurate visual cues revealed repetition‐induced anterograde interference. Their participants were instructed to move as quickly and accurately as possible to their targets (15‐mm radius). These high demands on speed and accuracy might have amplified interference effect (Pashler and Baylis 1991). In contrast, our reach‐to‐button task, which had a controlled reach time of 1 s (conceivably sufficient for planning digit position during this time, e.g., Vaidya et al. 2015) with minimal accuracy demands, did not show repetition‐induced anterograde interference. This suggests that task demands, like speed and accuracy, may play an additional critical role in susceptibility to interference. Finally, the additional design features of our task, such as similar digit positioning and consistent hand trajectories between left‐ and right‐sided button presses and the spatial proximity of closely spaced targets, might reduce the likelihood of forming distinct or competing sensorimotor memories, thereby minimizing susceptibility to repetition‐induced anterograde interference.

Our findings also indicate that repetition‐induced anterograde interference was not modulated by the number of post‐switch trials (matched vs. unmatched with pre‐switch trial numbers), suggesting that time, familiarity, or practice with post‐switch conditions had no effect on the extent of repetition‐induced anterograde interference. The design and comparisons between matched and unmatched post‐switch trials control for the total amount of exposure participants have to each configuration of the object. Asymmetries in exposure could influence post‐switch interference, as neural representations of motor plans can change with experience (Roth and Ding 2024). The lack of a difference between these matched and unmatched conditions would suggest that features of neural representations that depend on exposure are not a source of interference or different neural representations that depend on exposure with the post‐switch action plan. Furthermore, these secondary results showing no differences between matched and unmatched conditions are consistent with the findings of Hadjiosif et al. (2023), who reported that the performance of healthy controls was not significantly affected by longer ITIs, indicating that factors like task duration or timing of the ITIs did not influence the level of performance retention in controls. Since no significant performance differences were observed between our matched and unmatched conditions, it is unlikely that the time spent performing pre‐ and post‐switch lifts was the primary factor driving the anterograde interference observed. Factors beyond task duration may play a more substantial role in repetition‐induced anterograde interference.

Compared to previous studies using the same CoM‐switching paradigm (e.g., Fu et al. 2014; Schneider and Hermsdörfer 2022), we observed smaller torque errors following the switch. For example, Fu et al. (2014) and Schneider and Hermsdörfer (2022) reported stronger negative transfer (i.e., torque is in the opposite direction as the ideal M_Com_) after eight pre‐switch trials. Our data show partial transfer (i.e., torque is in the same direction as the ideal M_Com_, but not at the correct magnitude) when the CoM switch occurred after only one, three, or five pre‐switch trials, with a magnifying decline in the extent of transfer with increasing pre‐switch exposure. Together, these results suggest that increasing repetition increases interference.

Notably, repetition‐induced anterograde interference was particularly pronounced in the component of torque generated by the product of grip force and digit position configuration. This suggests that once a digit position pattern is learned—despite slight variations from trial to trial—it may become more resistant to change under more drastically different task demands (e.g., CoM switch). Building on findings by Zhang et al. (2010), who showed that digit position configuration is learned more quickly than the lift force partitioning, our results suggest that this rapid acquisition, while efficient, may come at a cost of flexibility, making the digit position component of torque generation more susceptible to the deleterious effects of repetition‐induced anterograde interference.

In this study, we found that repetition‐induced anterograde interference was in the visually incongruent object‐use task. This result supports prior findings that repetition‐induced interference arises in object manipulation, particularly when visual feedback is unhelpful (Salimi et al. 2000; Sager et al. 2024). However, it can also occur with reliable visual cues depending on task complexity (Fu and Santello 2012; Schneider et al. 2019, 2020) and has been observed in reach‐to‐target tasks with speed and accuracy demands (Verstynen and Sabes 2011). These findings suggest that repetition‐induced anterograde interference is driven by factors like sensory uncertainty—whether visual incongruence or the need for speed—and the overgeneralization of sensorimotor memories in these contexts. This leads to rigid, stereotyped motor behaviors that impair adaptability. Our results indicate that while repetition can reinforce motor skills, excessive reliance on it under certain conditions may hinder motor flexibility. Future work should investigate specific task conditions, such as the balance of visual feedback, speed, and accuracy, that exacerbate repetition‐induced interference, in order to develop training protocols that promote flexibility without compromising task proficiency.

## Author Contributions

**Catherine Anne Sager:** conceptualization, data curation, formal analysis, investigation, project administration, visualization, writing – original draft, writing – review and editing. **Ian Greenhouse:** methodology, resources, visualization, writing – review and editing. **Michelle Marneweck:** conceptualization, data curation, formal analysis, funding acquisition, investigation, methodology, resources, supervision, visualization, writing – original draft, writing – review and editing.

## Conflicts of Interest

The authors declare no conflicts of interest.

## Peer Review

The peer review history for this article is available at https://www.webofscience.com/api/gateway/wos/peer‐review/10.1111/ejn.70155.

## Data Availability

All data will be made available upon reasonable request. Codes can be found on GitHub for public availability.
